# The effect of a monetary incentive on return of a postal health and development questionnaire: a randomised trial [ISRCTN53994660]

**DOI:** 10.1186/1472-6963-5-55

**Published:** 2005-08-18

**Authors:** Sara Kenyon, Katie Pike, David Jones, David Taylor, Alison Salt, Neil Marlow, Peter Brocklehurst

**Affiliations:** 1 Reproductive Sciences Section, Department of Cancer Studies and Molecular Medicine, University of Leicester, Leicester, UK; 2 Department of Epidemiology and Public Health, University of Leicester, Leicester, UK; 3 Paediatrics Department, Great Ormond Street Hospital for Sick Children, London, UK; 4 Department of Child Health, University of Nottingham, Nottingham, UK; 5 National Perinatal Epidemiology Unit, Oxford, UK

## Abstract

**Background:**

Postal questionnaires are widely used to collect data in healthcare research but a poor response rate may reduce the validity and reliability of results. There was a lack of evidence available relating to use of a monetary incentive to improve the response rate in the healthcare setting.

**Methods:**

The MRC ORACLE Children Study is assessing the health and development of nearly 9000 seven year old children whose mothers' joined the MRC ORACLE Trial. We carried out a randomised controlled trial of inclusion of monetary incentive (five pound voucher redeemable at many high street stores) with the reminder questionnaire to parents. This trial took place between April 2002 and November 2003. When the parents were sent the reminder questionnaire about their child's health and development they were randomly assigned by concealed computer-generated allocation stratified by week of birthday to receive a five pound voucher or no incentive. The population were 722 non-responders to the initial mailing of a 12-page questionnaire. Main outcome measures: Difference in response rate between the two groups.

**Results:**

Inclusion of the voucher with the reminder questionnaire resulted in a 11.7%(95% CI 4.7% to 18.6%) improvement in the response rate between the two groups.

**Conclusion:**

This improvement in response rate and hence the validity and reliability of results obtained appears to be justified ethically and financially.

## Background

Postal questionnaires are widely used to collect data in health research, but a poor response rate may reduce the validity and reliability of results. In a systematic review of randomised controlled trials of strategies to improve the response rate to postal questionnaires[1], a monetary reward had a significant effect on response. However, caution was attached to the interpretation of the findings in this review [2]. On further examination of the updated review 20% of the participants included in the analysis of the effect of inclusion of a monetary incentive in the final response came from healthcare settings [3] and none of the studies evaluated the use of monetary incentives for a postal questionnaire to collect data from a follow-up of a clinical trial. To evaluate the impact of such an intervention on response rate in such a setting we undertook a randomised trial.

The MRC ORACLE Children Study (MOCS) is following up nearly 9000 seven-year-old children whose mothers joined the MRC ORACLE Trial [4,5] which evaluated the use of antibiotics to improve neonatal outcome after preterm labour or preterm rupture of the membranes. This trial of a monetary reward to enhance response to a postal questionnaire was undertaken between April 2002 – November 2003. Research Ethics Committee approval was obtained from the West Midlands Multicentre Research Ethics Committee

The questionnaire itself is 12 pages long and A4 in size. It contains questions relating to the child's health and development using a mixture of validated tools and questions pertinent to the study. In designing the study we implemented many of the strategies believed to influence response rates to postal questionnaires [1,3]. The questionnaire itself is set out in a user friendly way and we have used coloured ink in its design. The letters accompanying the questionnaire are individualised to the child concerned and the parents are warned the questionnaire will be sent to them. The University in which MOCS is housed franks the envelope, the return envelope is stamped, and reminder letters include a questionnaire.

## Methods

**Figure 1:**
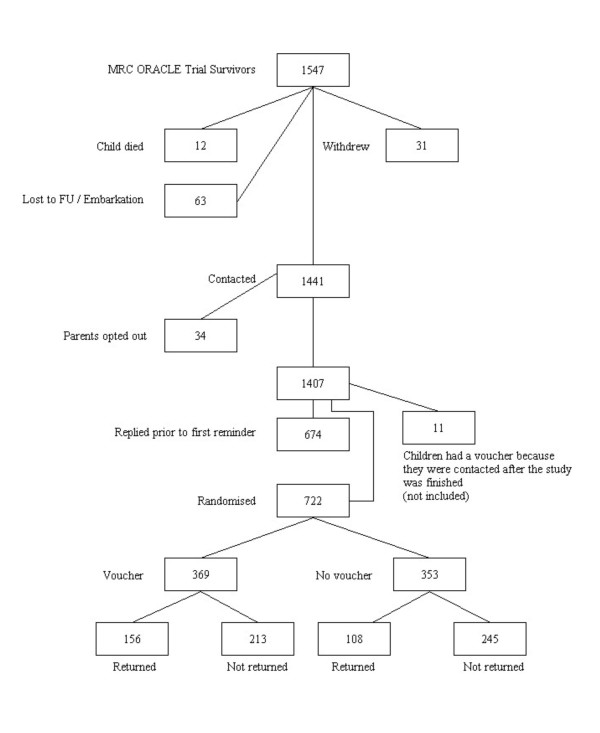
Consort flow diagram.

When a child in MOCS is seven years old the parents receive an information leaflet about the follow-up Study, and two weeks later a questionnaire about their child's health and development. Contact with parents has already been established prior to this. If no response is obtained the child's General Practitioner is contacted to check the child's address and ensure that contact would be appropriate. Six weeks after the first questionnaire, a reminder one is sent to those who have not responded. At this point the parents were randomly assigned by computer-generated allocation to receive a five-pound voucher (redeemable at many high street shops) with their mailed questionnaire or not (see Figure 1).

The sample size was predefined by the numbers not responding at this stage of the Study (i.e. approx 700). This yielded 80% power to detect an increased response from 10% to 18% or from 15% to 24% (at the 5% significance level).

## Results

Balance between voucher/not voucher groups on the main baseline covariates was good. 722 consecutive parents were randomly allocated to receive a voucher or not with the reminder questionnaire (see Table 1).

**Table 1:** Results of random allocation of voucher or not on response rate

	Voucher	No voucher
Questionnaire returned	156 (42.3%)	108 (30.6%)
Questionnaire not returned	213	245
Total	369	353

Inclusion of the voucher with the second questionnaire resulted in a 11.7% (95% CI 4.7% to 18.6%) improvement in the response rate between the two groups (χ^2 ^= 10.6, P = 0.001).

## Discussion

The inclusion of a five-pound voucher improved the proportion of questionnaires returned. MOCS will be completed in 2008 and a voucher is being sent to all parents with the reminder questionnaire. It is estimated that this will improve the response rate by 3% over the whole study, at a cost of £67 per additional questionnaire returned. This was calculated on the basis that vouchers will be sent to approximately 40% of parents (2842) and an additional 3% will return the questionnaire

This improvement in response rate, and hence of the validity and the reliability of results appears to be justified ethically and financially. This is particularly relevant in the follow up of children as there is some evidence [6] of raised levels of adverse outcomes in difficult to follow-up children.

## Competing interests

The author(s) declare that they have no competing interests.

## Contributors

SK and PB designed the trial. DJ and KP carried out the analysis. NM, AS, DT contributed to conception, design and interpretation. All authors read and approved the trial manuscript.

## Pre-publication history

The pre-publication history for this paper can be accessed here:


